# CTGF/CCN2 Postconditioning Increases Tolerance of Murine Hearts towards Ischemia-Reperfusion Injury

**DOI:** 10.1371/journal.pone.0149000

**Published:** 2016-02-12

**Authors:** Ole Jørgen Kaasbøll, Ingvild Tronstad Moe, Mohammad Shakil Ahmed, Espen Stang, Else Marie Valbjørn Hagelin, Håvard Attramadal

**Affiliations:** 1 Institute for Surgical Research, Oslo University Hospital and University of Oslo, Oslo, Norway; 2 Center for Heart Failure Research, University of Oslo, Oslo, Norway; 3 Dept. of Pathology, Oslo University Hospital, Oslo, Norway; Virginia Commonwealth University, UNITED STATES

## Abstract

**Background and Purpose:**

Previous studies of ischemia-reperfusion injury (IRI) in hearts from mice with cardiac-restricted overexpression of CCN2 have shown that CCN2 increases tolerance towards IRI. The objectives of this study were to investigate to what extent post-ischemic administration of recombinant human CCN2 (rhCCN2) would limit infarct size and improve functional recovery and what signaling pathways are involved.

**Experimental Approach:**

Isolated mice hearts were perfused ad modum Langendorff, subjected to no-flow, global ischemia, and subsequently, exposed to mammalian cell derived, full-length (38-40kDa) rhCCN2 (250 nM) or vehicle during the first 15 min of a 60 min reperfusion period.

**Key Results:**

Post-ischemic administration of rhCCN2 resulted in attenuation of infarct size from 58 ± 4% to 34 ± 2% (p < 0.001) which was abrogated by concomitant administration of the PI3 kinase inhibitor LY294002 (45 ± 3% vs. 50 ± 3%, ns). In congruence with reduction of infarct size rhCCN2 also improved recovery of left ventricular developed pressure (p < 0.05). Western blot analyses of extracts of ex vivo-perfused murine hearts also revealed that rhCCN2 evoked concentration-dependent increase of cardiac phospho-GSK3β (serine-9) contents.

**Conclusions and Implications:**

We demonstrate that post-ischemic administration of rhCCN2 increases the tolerance of ex vivo-perfused murine hearts to IRI. Mechanistically, this postconditioning effect of rhCCN2 appeared to be mediated by activation of the reperfusion injury salvage kinase pathway as demonstrated by sensitivity to PI3 kinase inhibition and increased CCN2-induced phosphorylation of GSK3β (Ser-9). Thus, the rationale for testing rhCCN2-mediated post-ischemic conditioning of the heart in more complex models is established.

## Introduction

Ischemic heart disease is the major cause of mortality in the USA and in the world at large [1]. Although the mortality of ischemic heart disease has declined during the last three decades in Western affluent countries [2], heart failure presenting many years after the index event (myocardial infarction) plays an increasing contribution to the mortality of ischemic heart disease in demographics with rising numbers of elderly [3, 4]. Thus, there is a demand for new treatment modalities that reduces myocardial tissue loss in acute coronary thrombosis.

One of the strategies for alleviating the burden of ischemic heart disease aims at minimizing infarct size in acute coronary syndromes. The current treatment of choice for limiting infarct size in patients with ST-segment myocardial infarction is rapid myocardial reperfusion using percutaneous coronary intervention (PCI). However, restoring blood flow to ischemic tissue may itself inflict serious damage. Experimental studies indicate that such damage, termed ischemia-reperfusion injury (IRI) may account for up to 50% of the ultimate infarct size following acute coronary thrombosis [5]. Ever since the discovery of the potential for reducing IRI by short cycles of ischemia prior to a major ischemic event, i.e. ischemic preconditioning [6], in 1986, substantial research efforts have focused on elucidating the signaling mechanisms that confer myocardial salvage from IRI, and to what extent the salutary effects could be mimicked by pharmacologic substances. These efforts led to the discovery of glycogen synthase kinase-3β (GSK3β) and the mitochondrial permeability transition pore as the points of convergence of many signaling pathways that increase tolerance toward IRI [7]. Several substances that increase the tolerance toward IRI of the heart in experimental model systems have been identified when administered before the ischemic event (i.e. pharmacologic preconditioning) [8], or for some, even when first administered upon reperfusion (i.e. pharmacologic postconditioning) [9]. The latter would be the clinically most relevant cardioprotective strategy, since coronary thrombosis and acute myocardial infarction cannot be predicted with certainty and, thus, therapy can first be instituted after the ischemic event has occurred. Yet, experimental evidence of ischemic postconditioning or pharmacologic postconditioning in animal models of ischemia-reperfusion injury have not yet translated into novel therapy that reduces infarct size in patients with acute coronary syndromes [10, 11]. However, promise still prevails for pharmacologic postconditioning. Recently, a multicenter study reported reduced infarct size in STEMI patients that received the β1-adrenergic receptor antagonist metoprolol immediately before reperfusion (PCI) [12].

Previously, we have shown that transgenic mice with cardiac-restricted overexpression of rat CCN2 (Tg-CCN2 mice) exhibits increased tolerance to IRI *in vivo* upon transient occlusion of the left anterior descending coronary artery [13]. It was also demonstrated that recombinant, human CCN2 (rhCCN2) was able to recapitulate the cardioprotective phenotype when Langendorff-perfused hearts were exposed to rhCCN2 before the ischemic event [13]. Furthermore, the cardioprotective action of CCN2 was shown to be conferred via the PI3K-AKT-GSK3β phosphokinase cascade [13, 14]. This phosphokinase cascade has also become to be known as the Reperfusion-Injury-Salvage-Kinase (RISK) pathway, a signaling pathway that several cardioprotective compounds feed into [15,16].

In the context of several unsuccessful attempts of translating preclinical findings to the clinic, we decided to follow the strategy put forth by the Hatter Working Group [17], where the need for thorough experimental research with progressively more complex models approaching the clinical settings of unanticipated ischemia was emphasized before proceeding to clinical testing. Hence, we aimed to test the hypothesis that post-ischemic administration of rhCCN2 may increase tolerance towards ischemia-reperfusion injury and reduce infarct size. As this was a proof-of-principle study, and pharmacokinetic data for *in vivo* administration of rhCCN2 were not available, we decided on the isolated, *ex vivo*-perfused heart model, which allowed for precise control of delivery of rhCCN2 during the first critical minutes of reperfusion during which myocardial necrosis develops [18]. In summary our findings demonstrate that post-ischemic administration of rhCCN2 in an isolated heart system attenuates infarct size and improves post-ischemic recovery of cardiac function.

## Materials and Methods

### Animals

All animal studies were performed in accordance with the *Guide for the Care and Use of Laboratory Animals* published by the National Institutes of Health **(NIH** Pub. No. 85–23, revised 2010), and were approved by the national board for animal research, the Norwegian Animal Research Authority (permit number: 3343). The animals used were 14–16 weeks old male C57BL/6JBomTac mice (Taconic), which were housed individually with ad libitum access to food and water for one week before the experiments.

### Materials

Recombinant human CCN2 (rhCCN2) was contract purified by EMP Genetech, Ingolstadt, Germany, as previously described [19]. Briefly, secreted, full-length (38–40 kDa) recombinant CCN2 was purified from the cell culture medium of HEK293 cells (adapted for suspension culture) stably transfected with human CCN2 cDNA containing the entire open reading frame. RhCCN2was purified by sequential heparin-Sepharose and Superose gel exclusion chromatography, dialyzed against phosphate-buffered saline (without Ca^2+^ and Mg^2+^ salts), and concentrated in Centriprep ultrafiltration device to approximately 0.3 μg/ml. Analysis of the purified protein by SDS-PAGE and subsequent staining of the gel with Coomassie brilliant blue or preparation for Western blot analysis, revealed that rhCCN2 migrated according to the predicted molecular mass of the full-length protein. rhCCN2 migrated as two discrete bands with molecular mass around 38–40 kDa previously reported to represent glycosylated and non-glycosylated forms [14, 20]. RhCCN2 in phosphate-buffered saline (without Ca^2+^ and Mg^2+^ salts) was added to the perfusion buffer or cell culture as indicated. *Ex vivo* perfusion of the hearts was performed with the Krebs Henseleit perfusion buffer (KHB) containing 118.5 mM NaCl, 25.0 mM NaHCO_3_, 4.7 mM KCl, 1.2 mM KH_2_PO_4_, 1.2 mM MgSO_4_, 11.0 mM glucose, 1.9 mM CaCl_2_ (all chemicals were of analytical grade from Merck KGaA, Germany). Other chemicals used were the phosphoinositide 3-kinase (PI3K) inhibitor LY294002 (Tocris Bioscience, Bristol, UK) and 2,3,5- triphenyltetrazolium chloride (TTC) (Sigma-Aldrich, St. Louis, MO).

### Experimental protocols

**Fig 1:**
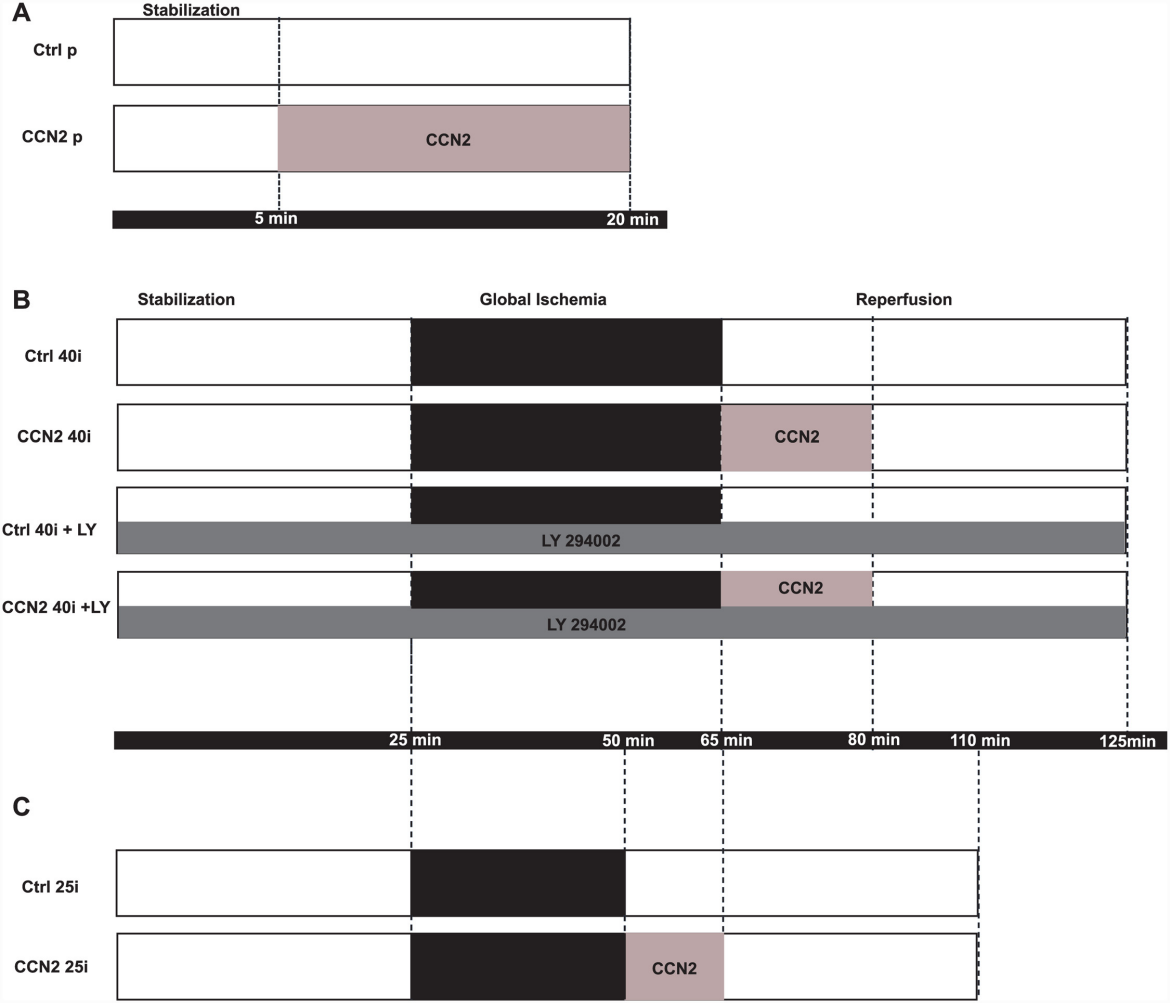
Schematic of experimental protocols. A. For phosphoprotein studies hearts were perfused with Krebs Henseleit buffer at 37°C in Langendorff setups. The perfused hearts were allowed to stabilize for 5 min before perfusion for 15 min without (Ctrl p) or with rhCCN2 (CCN2 p; 250 nM). B and C. For the ischemia-reperfusion studies Langendorff-perfused hearts were allowed to stabilize for 25 min before start of no-flow global ischemia. No-flow ischemia was maintained for 40 min (Ctrl 40i, CCN2 40i, Ctrl 40i +LY and CCN2 40i +LY) or 25 min (Ctrl 25i and CCN2 25i). In the CCN2 40i, CCN2 40i + LY and CCN2 25i the first 15 min of the 60 min reperfusion were in the presence of 250nM recombinant human CCN2. In the Ctrl 40i + LY and CCN2 40i + LY the entire perfusion was performed in the presence of 50μM LY294002.

First, pilot experiments were performed to determine the efficacy and potency of rhCCN2-stimulated activation of the PI3K-GSK3β phosphokinase cascade in primary cultures of adult cardiac myocytes and in ex vivo-perfused mouse hearts. Accordingly, primary adult cardiac myocytes and KHB-perfused hearts were exposed to increasing concentrations of rhCCN2 (15 min, 37°C) and rhCCN2-stimulated phospho-GSK3β (Ser-9) were subsequently assessed as described below. Based on these experiments a concentration of rhCCN2 that was submaximal (250 nM), yet elicited robust increase of phospho-GSK3β levels was chosen for the subsequent investigation of the postconditioning capacity of rhCCN2. This concentration of rhCCN2 was also in accordance with previously recorded efficacies of rhCCN2 in primary cardiac myocytes and rat2 fibroblasts [14]. In order to investigate to what extent rhCCN2 affords cardioprotection in ex vivo-perfused hearts when administered during the immediate reperfusion of the heart following ischemia (postconditioning), hearts were perfused ad modum Langendorff under constant perfusion pressure (80 mmHg). After mounting the hearts in the Langendorff perfusion mode, all hearts were allowed a 25 min period of stabilization before initiation of 40 or 25 min of no-flow, global ischemia at 37°C, followed by a reperfusion period of 60 min. The hemodynamic recordings after the stabilization period are depicted as baseline values. The experimental protocols are schematically illustrated in Fig 1. To investigate the potential for rhCCN2 to reduce infarct size after ischemia, pairs of hearts subjected to 40 min of no-flow global ischemia were randomized to reperfusion with either KHB (Ctrl, 40i), or KHB containing 250 nM rhCCN2 (CCN2 40i) (N = 11 pairs). RhCCN2 was included in the KHB buffer for the CCN2 40i hearts during the first 15 min of reperfusion. To investigate to what extent the cardioprotective capacity of rhCCN2 was dependent on PI3K, the canonical upstream activator of the Akt-GSK3β phosphokinase cascade, a new set of ex vivo ischemia-reperfusion experiments were performed in which the PI3K inhibitor LY294002 (50 μM) was included in the perfusion buffer and the hearts were exposed to the absence (Ctrl40i + LY) or presence (CCN240i + LY) of rhCCN2 (250 nM) during the first 15 min of reperfusion (N = 11 pairs). As the critical parameter of the cardioprotective potential of post-ischemically administered rhCCN2 was infarct size, the protocols for the aforementioned experimental groups were designed to generate large infarctions (40 min of no-flow ischemia) in order to more readily reveal a cardioprotective effect of rhCCN2. However, such a protocol rendered limited potential for rhCCN2 to improve post-ischemic cardiac function. Thus, in order to assess to what extent post-ischemic exposure to rhCCN2 would also improve contractile function, another set of experiments was performed with shorter ischemia time (25 min) before reperfusion in the absence (Ctrl 25i) or presence (CCN2 25i) of rhCCN2 (N = 10 pairs).

### Isolated heart preparation

The mice were anesthetized with an intraperitoneal injection of sodium pentobarbitone (180 mg/kg) mixed with heparin (4500 IU/kg). Following confirmation of adequate anaesthesia (absence of the pedal withdrawal reflex), the hearts were rapidly excised, cannulated and perfused with KHB saturated with 95% O_2_ / 5% CO_2_ gas mixture, in a Langendorff apparatus at 37°C, using constant perfusion pressure of 80 mmHg, as described in detail previously [21]. To avoid the confounding effects of time of day variations in ischemia tolerance among the control hearts and the CCN2-exposed hearts [22], all experiments were performed pairwise in two parallel Langendorff setups with a shared KHB reservoir. Such an experimental design reduces introduction of unwanted noise in the results, and allows for pairwise comparison between a set of control hearts and intervention hearts.

Left ventricular end-systolic pressure (LVSP) and left ventricular end-diastolic pressure (LVEDP) were acquired by a fluid-filled balloon made of cling film positioned in the intraventricular cavity and connected to a pressure transducer, as described previously [21], while left ventricular developed pressure (LVDP = LVSP—LVEDP) and heart rate were calculated (HR: analysis of peak-to-peak intervals of LVSP). The signals from the pressure transducers were amplified and continuously recorded by a PowerLab—LabChart 7.2 data acquisition system (all components from ADInstruments Ltd, Oxford, UK). During the stabilization period, the intraventricular balloons were inflated until LVEDPs were approximately 5 mmHg, and the balloon volumes were not altered thereafter.

At the end of the 25 min stabilization period, pairs of hearts among which at least one of the hearts did not conform to the inclusion criteria (LVSP ≥ 60 mmHg, HR ≥ 170 beats per min (bpm), were excluded from the study. Included pairs were subjected to no-flow, global ischemia according to the experimental protocols followed by 60 minutes of reperfusion.

### Determination of infarct size

Determination of infarct size (area of necrosis) was performed by analysis of serial sections of the heart stained with TTC (viability stain) as described previously [13]. Briefly, at the end of the reperfusion period, 1 mm thick sections (slices) of the heart, perpendicular to its long axis, were prepared using a 1 mm heart matrix (South Point Surgical, Fl, USA), giving approximately 7 slices per heart. The heart slices were incubated in PBS containing 1% TTC solution for 18 min at 37°C, followed by one hour in 4% paraformaldehyde in PBS, and kept overnight in PBS. Digital images of the heart slices were obtained by high resolution scanning under transilluminating light (Epson Perfection V750 Pro), and infarct size (area of necrosis; non-stained area) was determined by digital planimetry using Adobe Photoshop CS3 by an investigator blinded to the experimental groups. Infarct size was provided as percentage of total heart slice area (summation of all slices of the heart), as previously described [13].

### Phosphoprotein analysis of isolated heart extracts

Hearts were perfused ad modum Langendorff with KHB at 37°C in the absence or presence of increasing concentrations of rhCCN2 in order to assess to what extent CCN2 elicited activation of the PI3K–GSK3β pathway. Following perfusion of the hearts in the absence or presence of rhCCN2 for 15 min, the hearts were rapidly removed from perfusion, snap-frozen in liquid nitrogen, and stored at -80°C. The myocardial tissue samples were homogenized and solubilized at 4°C in buffer containing 10mM Tris-HCl (pH 7.4), 1% SDS, 10mM NaF and 2mM Na_3_VO_4_, denatured in Laemmli buffer, separated on a 12% SDS-PAGE, and electroblotted onto a PVDF membrane. The membrane was blocked in Tris-buffered saline (TBS) with 0.1% Tween-20 and 5% non-fat dry milk before incubation with primary antibody (phosphoserine-9-specific anti-GSK3β, #9336 and total anti-GSK3β, #9315; Cell Signaling Technology, Inc., MA,USA) in TBS containing 0.1% Tween-20 and 5% BSA) and, subsequently, horseradish peroxidase-conjugated secondary antibody. The immunoreactive bands were visualized with the chemiluminescent substrate LumiGLO Reserve (KPL, Gaithersburg, MD, USA).

### Cardiac myocyte isolation and maintenance

Primary, adult cardiac myocytes were isolated from murine hearts and maintained in culture as described previously [23]. Briefly, the aorta of murine hearts was cannulated and the hearts were perfused with collagenase type 2. Cardiac myocytes were separated from non-myocytes by centrifugation, before calcium was gradually reintroduced to a final of 1.2 mM and the myocytes were plated on laminin-coated wells in minimum essential medium (MEM) with Hanks’ balanced salts (HBSS) and supplemented with 10% fetal calf serum and 1 mM 2,3-butanedione monoxime. After 2 hours the plating medium was replaced with culture medium (MEM with HBSS) containing 0.1% bovine serum albumin and 1 mM 2,3-butanedione monoxime. The cardiac myocytes were maintained in a humidified atmosphere with 2% CO_2_. On the day of the experiment (24hours after plating in cell culture dishes) triplicate wells of cardiac myocytes were stimulated with increasing concentrations of rhCCN2 for 30min and subsequently harvested for analysis of phospho-GSK3β (Ser-9) contents. The experiment was repeated three times on cardiac myocytes from different mice.

### Phosphoprotein analysis of isolated cardiac myocyte extracts

Cardiac myocytes stimulated with rhCCN2 for 30min were harvested and protein was extracted with the Bio-Plex cell lysis buffer (Bio-Rad Laboratories Inc., CA, USA). Protein concentrations were measured with the Pierce^™^ BCA kit (Thermo Fisher Scientific, Rockford, Il, USA), and equal amount of protein was analyzed by luminex bead assay using antibody-coupled beads reactive against phospho-GSK3β (Ser-9) (Cat# 171V23318) and the BioPlex 200 recorder according to manufacturer’s instructions (Bio-Rad Laboratories Inc., CA, USA).

### Statistical analysis

Infarct size was subjected to statistical analysis using a two-tailed student’s t-test for paired values. The hemodynamical parameters were normalised to the baseline values, and compared by a repeated measures ANOVA. All values are given as mean ± SEM unless otherwise indicated. P-values < 0.05 were considered significant. All statistical analyses were performed with GraphPad Prism 5.0 (GraphPad Software Inc, CA, USA).

## Results

### Analysis of CCN2-stimulated myocardial phospho-GSK3β (Ser-9) levels

**Fig 2:**
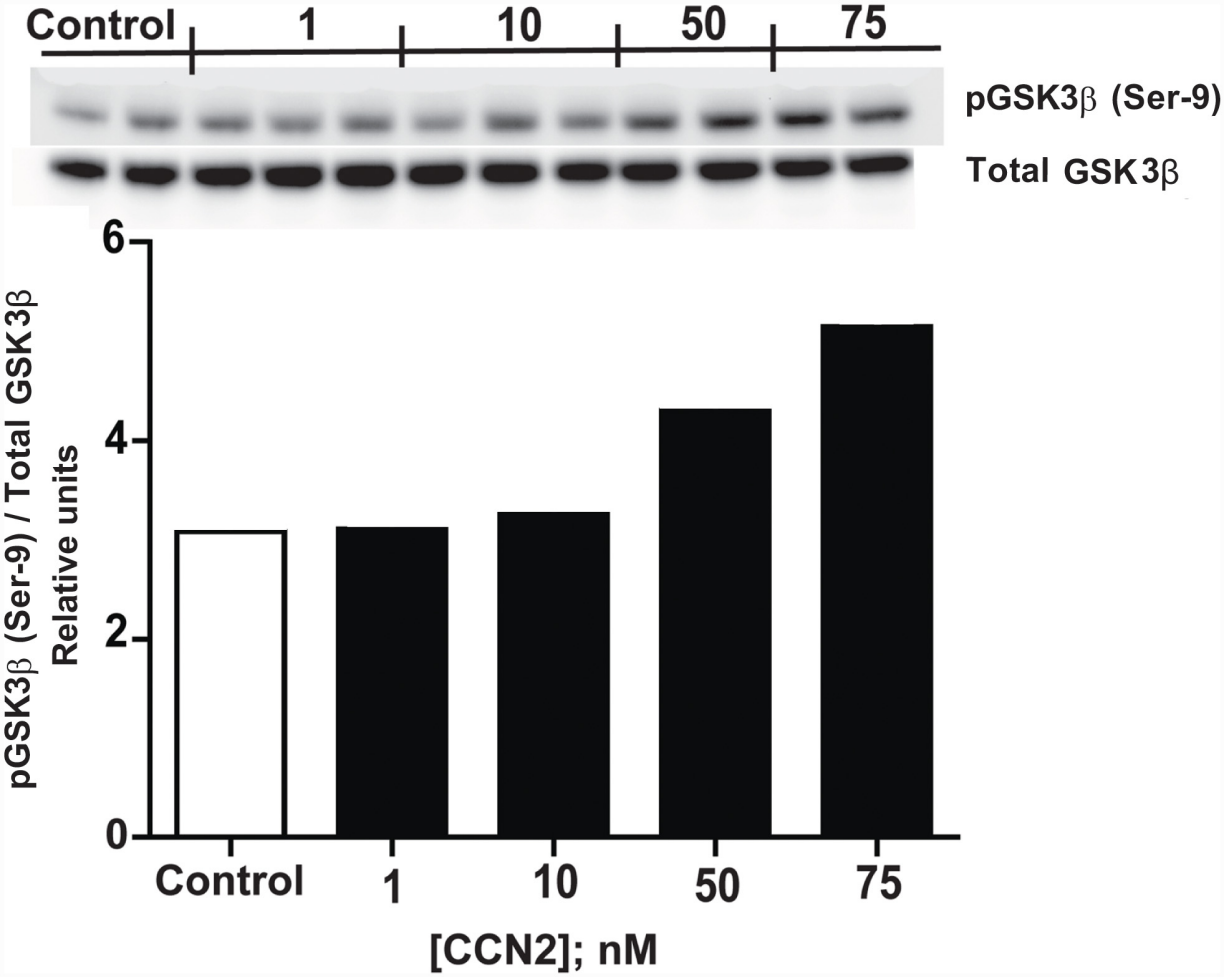
Phospho-GSK3β (Ser 9) contents in rhCCN2 stimulated hearts. Western blot analysis of phospho-GSK3β (Ser-9) contents in extracts of murine hearts following ex vivo perfusion of the hearts (Langendorff-perfusion) in the absence or presence of increasing concentrations of recombinant human CCN2. Each lane on the Western blot represents tissue from individual hearts. The histogram demonstrates group means of phospho-GSK3β contents assessed by densitometric analysis of the immunoreactive bands on the Western blot. The phospho-GSK3β (Ser-9) contents are displayed relative to total immunoreactive GSK3β contents. (Each group is mean of 2–3 *ex vivo*-perfused hearts)

**Fig 3:**
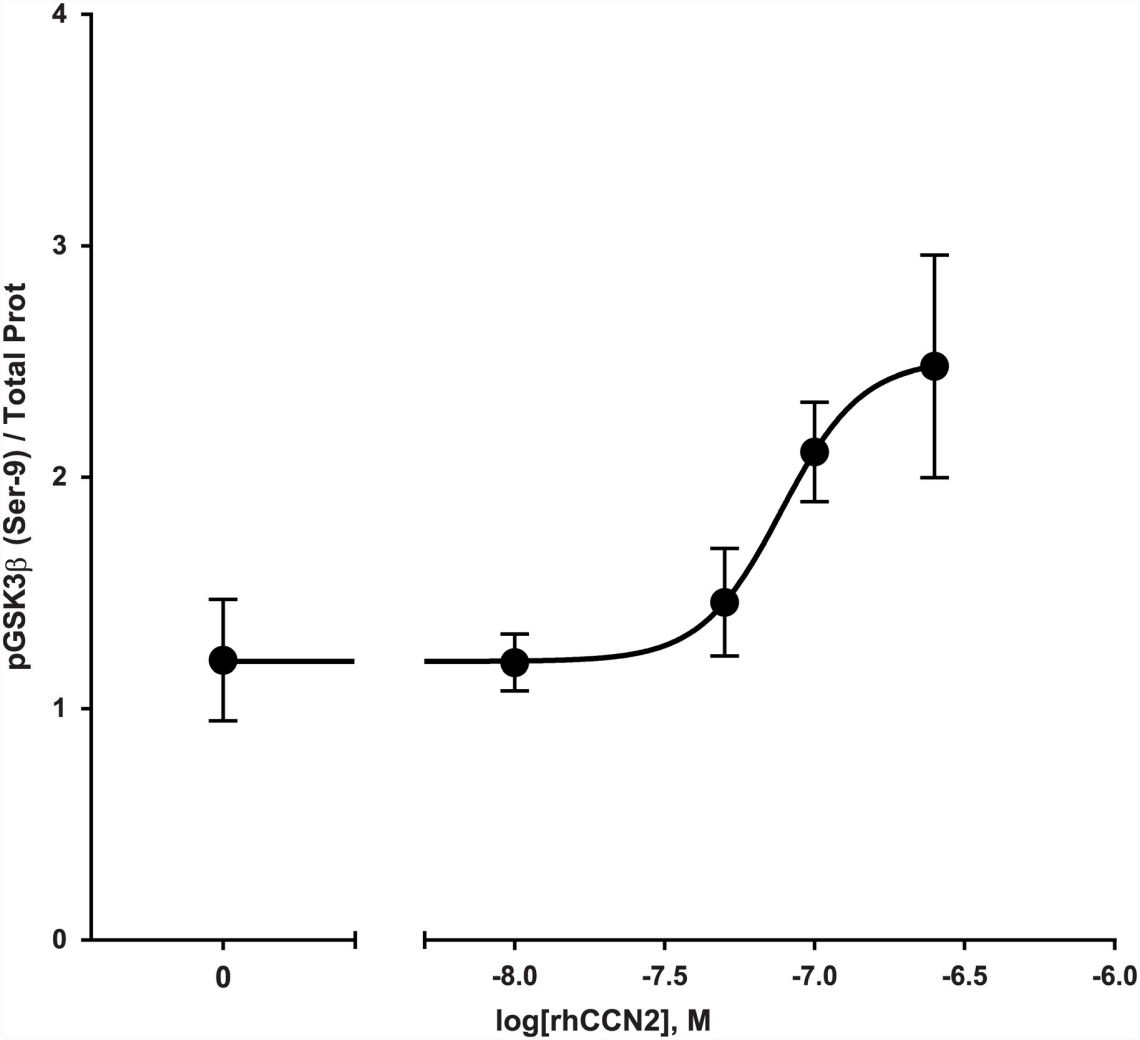
Phospho-GSK3β (Ser 9) contents in rhCCN2-stimulated cardiac myocytes. Concentration-effect curve of rhCCN2-stimulated phospho-GSK3β (Ser-9) contents in cardiac myocytes. Isolated adult cardiac myocytes were stimulated for 30 min in the presence of increasing concentrations of rhCCN2 before the incubation was stopped, protein was extracted and analyzed for phosphorylation of GSK3β (Ser-9) by ELISA using Luminex technology (Bio-Plex phospho-GSK-3β (Ser-9) assay, Bio-Rad Laboratories, Inc, CA, USA) according to the manufacturer’s instructions. The graph depicts a representative experiment mean±SD from triplicate wells, with the curve fitted by a non-linear, least squares method with variable slope, R^2^ = 0.83 (Graphpad Prism 5.0). The experiment was repeated three times.

Western blot analyses of extracts of murine hearts perfused with KHB *ex vivo* in the presence of increasing concentrations of rhCCN2 was analyzed for phosphorylation of GSK3β at serine-9, and a concentration-dependent phosphorylation trend was observed (Fig 2). This concentration-effect relationship between rhCCN2 and phosphorylation of GSK3β (Ser 9) in ex vivo-perfused hearts was corroborated by similar findings in primary adult mouse cardiac myocytes stimulated in the presence of increasing concentrations of rhCCN2 (Fig 3).

### Infarct size

**Fig 4:**
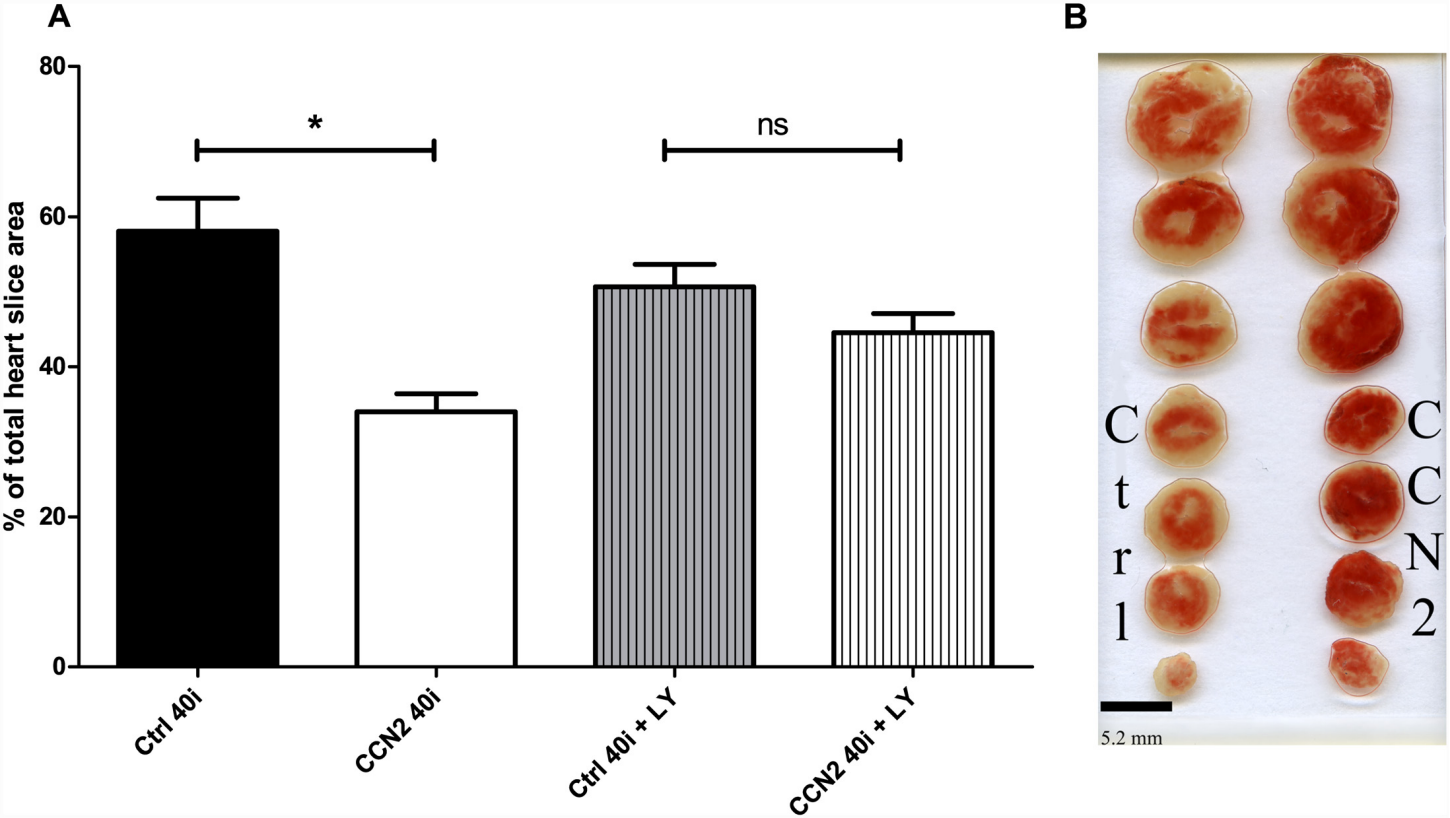
Infarct sizes in hearts subjected to rhCCN2 postconditioning. A: Morphometric analysis of infarct sizes as percentages of total heart slice area. B: Representative example of TTC-stained hearts from the groups Ctrl 40i and CCN2 40i. Data are mean ± SEM of N = 11 in each group. * *P*<0.05

After 40 min of no-flow ischemia, post-ischemic administration rhCCN2 during the first 15 min of reperfusion reduced global infarct size from 58 ± 4% to 34 ± 2% (p < 0.001) (Fig 4A and 4B). In the presence of LY294002, the rhCCN2-engendered reduction of infarct size was abolished (45 ± 3% vs 51 ± 3%, ns). Reperfusion in the presence of rhCCN2 also reduced global infarct size after a shorter period (25 min) of ischemia (Ctrl 25i vs CCN2 25i; 46 ± 4% vs 33 ± 3% respectively, p < 0.05).

### Cardiac function

**Fig 5:**
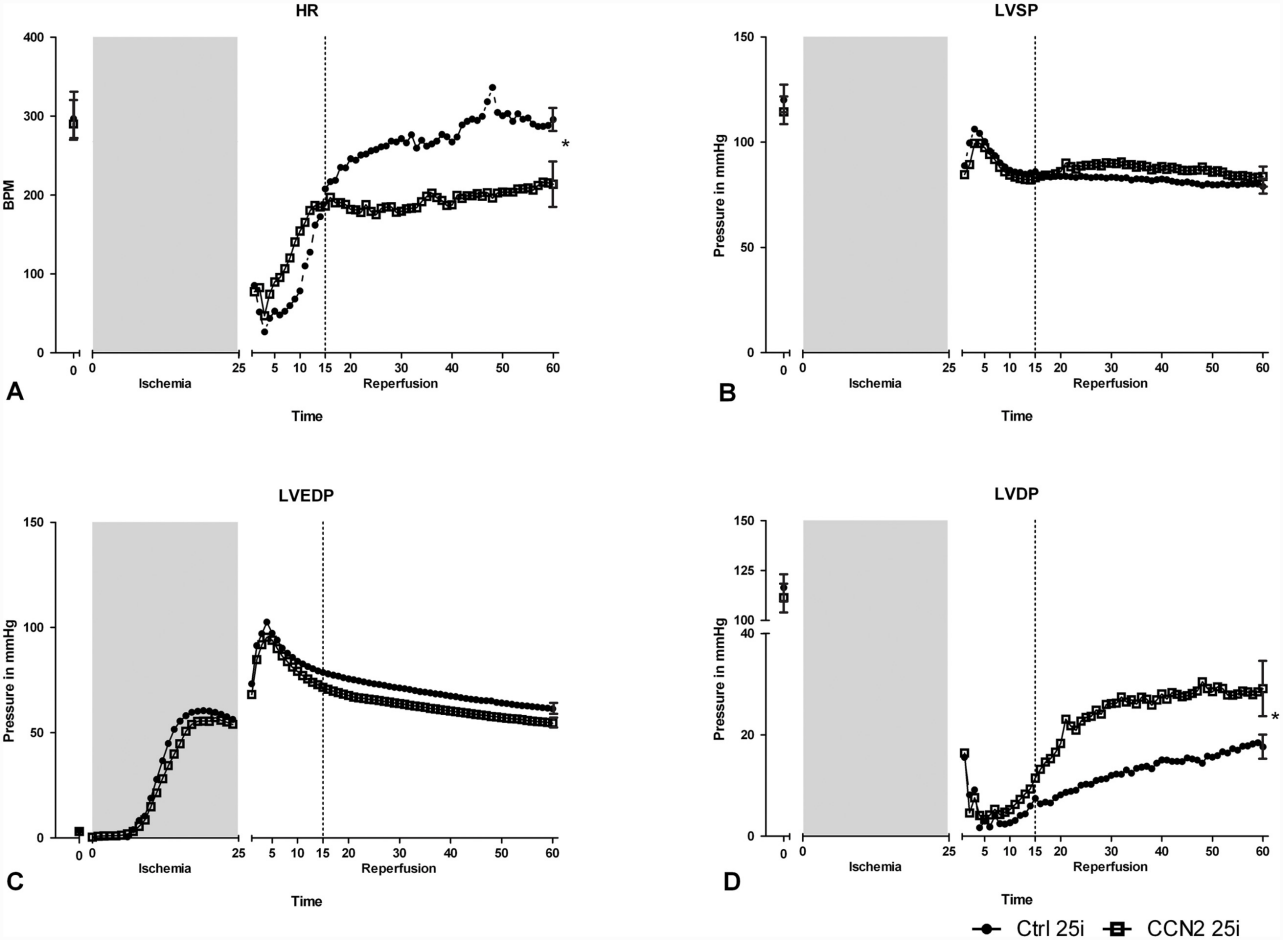
Cardiac function in hearts subjected to rhCCN2 postconditioning. Cardiac function of hearts subjected to 25 min of ischemia and subsequently perfused in the absence or presence of rhCCN2 (250 nM) during the first 15 min of reperfusion. The figure demonstrates recording of heart rate (A), left ventricular end-systolic pressure, LVSP (B), left ventricular end-diastolic pressure (LVEDP) (C), and left ventricular developed pressure, LVDP (LVSP—LVEDP) (D) at baseline and during reperfusion after ischemia. LVEDP (C), illustrates the “ischemic contracture” of the Langendorff global ischemia system during ischemia. Recovery of LVDP is significantly increased in the group receiving recombinant human CCN2 during reperfusion (CCN2 25i). Data are mean, ± SEM for the first and last data point of N = 10 in each group. * *P*<0.05.

Post-ischemic administration of rhCCN2 also improved recovery of cardiac function as revealed by the contractility surrogate LVDP (Ctrl 25i vs CCN2 25i, p < 0.05), as shown in Fig 5. LVDP was used instead of LV +(dP/dt)_max_ as an index of systolic cardiac function since the fluid-filled balloon in the LV cavity does not respond adequately to the rapid changes in LV volumes occurring at normal mouse heart rates (HR) to allow reliable recording of LV ±dP/dt [21]. The enhanced recovery of LVDP in hearts exposed to rhCCN2 during the initial phase of reperfusion may be due to the combined effects of a decrease of ischemic contracture (LVEDP) and an increase of LVSP, although neither of the latter alterations were found to be statistically significant.

Despite limited recovery of cardiac function in the hearts exposed to 40 min of ischemia, assessments of cardiac function in the experimental groups Ctrl 40i vs CCN2 40i and Ctrl 40i + LY vs CCN2 40i + LY revealed enhanced PI3K-dependent recovery of cardiac function after post-ischemic administration of rhCCN2. Thus, LVDP culminated significantly higher in the CCN2 40i group compared to the Ctrl 40i group (p < 0.01, S1 Fig). However, in the presence of LY294002, the post-ischemic improvement of LVDP afforded by rhCCN2 was abolished (S1 Fig).

Post-ischemic administration of rhCCN2 also affected the HR causing an apparently accelerated recovery, both in the series exposed to 25 min of ischemia and in those 40 min of ischemia, Fig 5A. However, these effects of rhCCN2 were not sustained after the administration of rhCCN2 was stopped (Ctrl 40i vs CCN2 40i). Rather, in the experiments where 25 min of ischemia were employed, the HR of the Ctrl 25i group surpassed that of the CCN2 25i group after administration of rhCCN2 was terminated. However, the latter courses of HRs did not appear to be a consistent finding as HRs of the Ctrl40i and CCN2 40i groups were not statistically different during the course after administration of rhCCN2 had been terminated.

## Discussion

In this work we demonstrate that post-ichemic administration of rhCCN2 reduces infarct size and improves functional recovery after ischemia-reperfusion injury. Mechanistically, we show that stimulation of isolated cardiac myocytes and perfusion of isolated hearts with rhCCN2 stimulates phosphorylation of GSK3β, and that addition of the PI3K inhibitor LY294002 abolishes the cardioprotective effects of rhCCN2. These findings imply that activation of the RISK pathway is the principal mediator of the postconditioning actions elicited by rhCCN2.

The ability of rhCCN2 to afford cardioprotection when administered during the immediate early phase of reperfusion not only demonstrates its potential as a cardioprotective agent, it also implies that CCN2-engendered signaling must activate more or less instantaneous effector mechanisms. This contention is based on previous observations demonstrating that ischemia-reperfusion injury that is amenable to salvage by a postconditioning agent is established during first minutes after initiation of reperfusion [5, 11], with cellular swelling and appearance of dense granular bodies in the mitochondria in the ischemic region evident as early as 2 minutes after onset of reperfusion and nearly universally present after 5–10 minutes of reperfusion [24]. Thus, the CCN2-mediated postconditioning is likely to be conferred by a rapid phosphokinase-regulated posttranslational effector mechanism rather than by a signaling mechanism depending on the successive induction of a transcriptome and ultimately a cardioprotective proteome. Yet, previous reports from our laboratory demonstrate that CCN2 also activates a cardiac transcriptome that may increase tolerance toward ischemia-reperfusion injury [13, 14]. Interestingly in this respect, the efficacy of the pharmacologic preconditioning or postconditioning actions of rhCCN2 elicited by short exposures was substantially less than that reported for ischemia-reperfusion injury of transgenic-CCN2 mice with cardiac-restricted overexpression of CCN2 [13]. The increased tolerance of transgenic-CCN2 mice towards ischemia-reperfusion injury was associated with induction of an explicit cardioprotective transcriptome putatively accounting for the more robust reduction of myocardial necrosis [13]. Despite these distinctive findings, it cannot be excluded that increased tolerance towards ischemia-reperfusion injury following even brief exposures to rhCCN2 may be partially dependent on rapid activation of a cardioprotective transcriptome.

Although our data do implicate the RISK pathway described by Yellon and Hausenloy [9] in CCN2-mediated postconditioning of the heart, the signaling mechanisms upstream of PI3K are poorly understood and a cognate receptor for CCN2 remains to be identified. As a secreted matricellular protein, CCN2 may either bind cell surface receptors on the plasma membrane or work by binding and modulating the function of other extracellular proteins [25]. In this study, we establish the ability of rhCCN2 to rapidly initiate signaling cascades in *ex vivo*-perfused hearts in the absence of blood, excluding the possibility of CCN2-mediated cardioprotection being dependent on blood-borne factors or blood-borne cells. However, CCN2 has been reported to bind several blood-borne or autocrine/paracrine factors; TGFβ [26], BMP-4 [26], BMP-2 [27], FGF-2 [28, 29], EGF [30, 31], IGF-2 [30, 31], IGF-1 [32, 33] and VEGF [34]. Also the closely related CCN family member, CCN1, has been described to interact with both TNFα [35] and neutrophils [36]. Thus, while the observations that rhCCN2 rapidly activates phosphokinase signaling by direct actions on target cells strongly support a receptor-mediated transmembrane signaling mechanism, indirect actions via binding of matrix-associated reservoirs of any of the above mentioned growth factors or cytokines cannot be excluded.

Previous reports from our laboratory and others have shown that the PI3K-AKT-GSK3β cascade is a prominent signaling pathway of CCN2 [13, 14, 37]. The ability of PI3K inhibition to abrogate the cardioprotective effects of post-ischemic administration of rhCCN2, both with regards to the limitation of infarct size and the improved functional recovery, clearly implicates PI3K as a necessary element of the intracellular signalling pathway of CCN2. This is further supported by the capacity of rhCCN2 to stimulate concentration-dependent phosphorylation of GSK3β (Ser9) in isolated adult cardiac myocytes as well as a trend for similar concentration-dependent increase of myocardial phospho-GSK3β (Ser-9) contents in isolated *ex* vivo-perfused hearts. The findings that both hearts from mice with cardiac-restricted overexpression of CCN2 as well as hearts perfused with recombinant human CCN2 before ischemia or during onset of reperfusion display similar cardiac phenotypes, i.e. increased tolerance to ischemia-reperfusion injury, support the notion of a uniform action of CCN2 in these model systems.

Tissue levels of CCN2 has been reported to be substantially increased in models of tissue damage (e.g. acute myocardial infarction) as well as in chronic inflammatory disorders with progressive fibrosis [38;39]. On the contrary, genetically-engineered mice with cardiac-restricted overexpression of CCN2 displayed phenotype with subtle increase of myocardial contents and no detectable impairment of cardiac function [13, 40–42]. Yet, studies of various cardiac disease models (myocardial infarction, chronic pressure overload) in mice with overexpression, knock-down or inhibition of CCN2 have reported divergent results regarding the role of CCN2 in maladaptive remodelling of the chronically overloaded heart [40–45]. To what extent these divergent findings are related to different aetiologies and pathophysiologic mechanisms of heart disease remain to be resolved. Thus, to what extent CCN2 is good or bad in chronic heart disease also remains to be settled. Early revascularization therapy following acute coronary thrombosis may save myocardial tissue from necrosis. In this respect, an overriding hypothesis is that substances or factors that may reduce reperfusion injury during the critical moments after revascularization therapy may reduce irreversible myocardial damage [11]. In this context, this study deals with the acute or short term-effect of CCN2 in salvage of myocardial tissue during reperfusion following ischemia. In the ischemia-reperfusion injury scenario specifically investigated in this study, the effects on IRI are due to rapid mechanisms, as discussed above, and the short-term administration of rhCCN2 are hence not likely to have lasting detrimental effects on cardiac physiology. Indeed, reduction of myocardial necrosis (infarct size) may limit later pathophysiologic remodelling and delay ventricular dilatation and onset of heart failure [46, 47].

Perfusion of the hearts in the presence of rhCCN2 also appeared to affect heart rate. The enhanced increase of heart rate during administration of rhCCN2 is most likely attributable to enhanced recovery of the ischemic myocardium and rescue of myocardial tissue. These findings are also consistent with the enhanced recovery of heart rate in hearts from Tg-CCN2 mice after no-flow ischemia of the heart [13]. The disparate effects of rhCCN2 on heart rate after perfusion with rhCCN2 in hearts exposed to 25 min of ischemia versus hearts exposed to 40 min of ischemia are difficult to interpret. Previous studies of ischemia-reperfusion injury of *ex vivo*-perfused murine hearts have to very limited degree provided attention to alterations of heart rate [48, 49]. Although we cannot rule out a direct effect of CCN2 on the HR, we have previously shown that administration of rhCCN2 to *ex vivo* perfused hearts immediately before onset of ischemia does not affect the HR after functional recovery has come to an end [13].

Several research groups have been working to identify autocrine/paracrine factors or signaling pathways capable of eliciting post-ischemic conditioning of the heart in order to identify targets amenable to pharmacologic intervention in acute coronary ischemia [11]. However, no pharmacologic compounds have yet successfully passed clinical testing in patients with acute coronary syndromes. The landmark study by Staat et al. [50], which demonstrated that ischemic postconditioning in order to alleviate reperfusion injury is a clinically relevant concept, clearly established the need to continue the search for a pharmacological agent capable of conferring post-conditioning of the heart. In this setting, the current study adds rhCCN2 to the list of substances that may be tested for clinical efficacy in pharmacologic postconditioning of coronary syndromes.

An obvious limitation of our study is the use of the *ex vivo* model of ischemia-reperfusion, as there are many other factors capable of influencing the outcome of an ischemia reperfusion event in the clinical setting which are not recapitulated by the reductionist Langendorff system, e.g. patients’ age, comorbidities, and polypharmacy. However, the choice of this model system was necessitated by the need to avoid the complications of infusion of a recombinant protein with undefined *in vivo* pharmacokinetics. Regardless of this limitation, we believe that together with the previous studies from our group, in which we demonstrate that transgenic mice overexpressing CCN2 attain smaller infarct size than wild type controls after exposure to an ischemia-reperfusion insult [13], and that isolated cardiac myocytes stimulated with rhCCN2 exhibit increased tolerance towards hypoxia-reoxygenation injury [14], this study clearly establishes CCN2 as a cardioprotective factor. Nevertheless, to proceed towards clinical testing of the cardioprotective properties of CCN2, the concept of CCN2-engendered post-ischemic conditioning of the heart will have to be tested *in vivo*, and preferably in a large animal model more closely resembling acute myocardial infarction in human patients.

In conclusion, this study can be considered a proof-of-principle study for the capacity of rhCCN2 to afford infarct size-limiting cardioprotection when administered post-ischemically, a scenario analogous to the clinical setting of revascularisation after acute myocardial ischemia.

## Supporting Information

S1 Fig Cardiac function in hearts subjected to rhCCN2 postconditioning after 40 min of ischemia. Cardiac function of hearts subjected to 40 min of ischemia and subsequently perfused in the absence or presence of rhCCN2 (250 nM) during the first 15 min of reperfusion. The figure demonstrates left ventricular developed pressure, LVDP (LVSP—LVEDP) at baseline and during reperfusion after ischemia. Recovery of LVDP is significantly increased in the group receiving recombinant human CCN2 (CCN2 40i) during reperfusion (A), while this effect is abolished by the presence of LY294002 (B). Data are mean, ± SEM for the first and last data point of N = 9–11 in each group. * *P*<0.05.
(EPS)

S1 File Raw data for Figs 3, 4A, 5 and S1 Fig. (PZF)

## Abbreviations

GSK3β: glycogen synthase kinase-3β
HR: heart rate
IGF-1: insulin-like growth factor 1
IRI: ischemia-reperfusion injury
KHB: Krebs Henseleit perfusion buffer
LVDP: left ventricular developed pressure
LVEDP: left ventricular end-diastolic pressure
LVSP: left ventricular end-systolic pressure
PI3K: phosphoinositide 3-kinase
rhCCN2: recombinant, human CCN2
RISK pathway: Reperfusion-Injury-Salvage-Kinase pathway
Tg-CCN2: transgenic mice with cardiac-restricted overexpression of rat CCN2
VEGF: vascular endothelial growth factor

## References

[1] Lozano R, Naghavi M, Foreman K, Lim S, Shibuya K, Aboyans V, et al Global and regional mortality from 235 causes of death for 20 age groups in 1990 and 2010: a systematic analysis for the Global Burden of Disease Study 2010. Lancet 2012 12 15;380(9859):2095–128. 10.1016/S0140-6736(12)61728-0 23245604 PMC10790329

[2] Moran AE, Forouzanfar MH, Roth GA, Mensah GA, Ezzati M, Murray CJ, et al Temporal trends in ischemic heart disease mortality in 21 world regions, 1980 to 2010: the global burden of disease 2010 study. Circulation 2014 4 8;129(14):1483–92. 10.1161/CIRCULATIONAHA.113.004042 24573352 PMC4181359

[3] Spoon DB, Psaltis PJ, Singh M, Holmes DR Jr., Gersh BJ, Rihal CS, et al Trends in cause of death after percutaneous coronary intervention. Circulation 2014 3 25;129(12):1286–94. 10.1161/CIRCULATIONAHA.113.006518 24515993

[4] Weir RA, McMurray JJ, Velazquez EJ. Epidemiology of heart failure and left ventricular systolic dysfunction after acute myocardial infarction: prevalence, clinical characteristics, and prognostic importance. Am J Cardiol 2006 5 22;97(10A):13F–25F. 16698331 10.1016/j.amjcard.2006.03.005

[5] Yellon DM, Hausenloy DJ. Myocardial reperfusion injury. N Engl J Med 2007 9 13;357(11):1121–35. 17855673 10.1056/NEJMra071667

[6] Murry CE, Jennings RB, Reimer KA. Preconditioning with ischemia: a delay of lethal cell injury in ischemic myocardium. Circulation 1986 11;74(5):1124–36. 3769170 10.1161/01.cir.74.5.1124

[7] Juhaszova M, Zorov DB, Kim SH, Pepe S, Fu Q, Fishbein KW, et al Glycogen synthase kinase-3beta mediates convergence of protection signaling to inhibit the mitochondrial permeability transition pore. J Clin Invest 2004 6;113(11):1535–49. 15173880 10.1172/JCI19906 PMC419483

[8] Liu GS, Thornton J, Van Winkle DM, Stanley AW, Olsson RA, Downey JM. Protection against infarction afforded by preconditioning is mediated by A1 adenosine receptors in rabbit heart. Circulation 1991 7;84(1):350–6. 2060105 10.1161/01.cir.84.1.350

[9] Hausenloy DJ, Yellon DM. New directions for protecting the heart against ischaemia-reperfusion injury: targeting the Reperfusion Injury Salvage Kinase (RISK)-pathway. Cardiovasc Res 2004 2 15;61(3):448–60. 14962476 10.1016/j.cardiores.2003.09.024

[10] Hausenloy DJ, Yellon DM. The therapeutic potential of ischemic conditioning: an update. Nat Rev Cardiol 2011 6 21. 10.1038/nrcardio.2011.85 21691310

[11] Hausenloy DJ, Yellon DM. Myocardial ischemia-reperfusion injury: a neglected therapeutic target. J Clin Invest 2013 1 2;123(1):92–100. 10.1172/JCI62874 23281415 PMC3533275

[12] Ibanez B, Macaya C, Sanchez-Brunete V, Pizarro G, Fernandez-Friera L, Mateos A, et al Effect of early metoprolol on infarct size in ST-segment-elevation myocardial infarction patients undergoing primary percutaneous coronary intervention: the Effect of Metoprolol in Cardioprotection During an Acute Myocardial Infarction (METOCARD-CNIC) trial. Circulation 2013 10 1;128(14):1495–503. 24002794 10.1161/CIRCULATIONAHA.113.003653

[13] Ahmed MS, Gravning J, Martinov VN, von Lueder TG, Edvardsen T, Czibik G, et al Mechanisms of novel cardioprotective functions of CCN2/CTGF in myocardial ischemia-reperfusion injury. Am J Physiol Heart Circ Physiol 2011 4;300(4):H1291–H1302. 10.1152/ajpheart.00604.2010 21186275

[14] Moe IT, Pham TA, Hagelin EM, Ahmed MS, Attramadal H. CCN2 exerts direct cytoprotective actions in adult cardiac myocytes by activation of the PI3-kinase/Akt/GSK-3beta signaling pathway. J Cell Commun Signal 2013 3;7(1):31–47. 10.1007/s12079-012-0183-1 23208610 PMC3590365

[15] Tsang A, Hausenloy DJ, Mocanu MM, Yellon DM. Postconditioning: a form of "modified reperfusion" protects the myocardium by activating the phosphatidylinositol 3-kinase-Akt pathway. Circ Res 2004 8 6;95(3):230–2. 15242972 10.1161/01.RES.0000138303.76488.fe

[16] Hausenloy DJ, Tsang A, Mocanu MM, Yellon DM. Ischemic preconditioning protects by activating prosurvival kinases at reperfusion. Am J Physiol Heart Circ Physiol 2005 2;288(2):H971–H976. 15358610 10.1152/ajpheart.00374.2004

[17] Hausenloy DJ, Baxter G, Bell R, Botker HE, Davidson SM, Downey J, et al Translating novel strategies for cardioprotection: the Hatter Workshop Recommendations. Basic Res Cardiol 2010 11;105(6):677–86. 10.1007/s00395-010-0121-4 20865418 PMC2965360

[18] Kloner RA, Ganote CE, Whalen DA Jr., Jennings RB. Effect of a transient period of ischemia on myocardial cells. II. Fine structure during the first few minutes of reflow. Am J Pathol 1974 3;74(3):399–422. 4814895 PMC1910797

[19] Ahmed MS, Øie E, Vinge LE, Yndestad A, Andersen G, Andersson Y, et al Connective tissue growth factor—a novel mediator of angiotensin II-stimulated cardiac fibroblast activation in heart failure in rats. J Mol Cell Cardiol 2004 3;36(3):393–404. 15010278 10.1016/j.yjmcc.2003.12.004

[20] Bohr W, Kupper M, Hoffmann K, Weiskirchen R. Recombinant expression, purification, and functional characterisation of connective tissue growth factor and nephroblastoma-overexpressed protein. PLoS One 2010;5(12):e16000 10.1371/journal.pone.0016000 21209863 PMC3012735

[21] Sutherland FJ, Shattock MJ, Baker KE, Hearse DJ. Mouse isolated perfused heart: characteristics and cautions. Clin Exp Pharmacol Physiol 2003 11;30(11):867–78. 14678252 10.1046/j.1440-1681.2003.03925.x

[22] Durgan DJ, Pulinilkunnil T, Villegas-Montoya C, Garvey ME, Frangogiannis NG, Michael LH, et al Short communication: ischemia/reperfusion tolerance is time-of-day-dependent: mediation by the cardiomyocyte circadian clock. Circ Res 2010 2 19;106(3):546–50. 20007913 10.1161/CIRCRESAHA.109.209346 PMC3021132

[23] O'Connell TD, Rodrigo MC, Simpson PC. Isolation and culture of adult mouse cardiac myocytes. Methods Mol Biol 2007;357:271–96. 17172694 10.1385/1-59745-214-9:271

[24] Kloner RA, Ganote CE, Whalen DA Jr., Jennings RB. Effect of a transient period of ischemia on myocardial cells. II. Fine structure during the first few minutes of reflow. Am J Pathol 1974 3;74(3):399–422. 4814895 PMC1910797

[25] Frangogiannis NG. Matricellular proteins in cardiac adaptation and disease. Physiol Rev 2012 4;92(2):635–88. 10.1152/physrev.00008.2011 22535894 PMC4411042

[26] Abreu JG, Ketpura NI, Reversade B, De Robertis EM. Connective-tissue growth factor (CTGF) modulates cell signalling by BMP and TGF-beta. Nat Cell Biol 2002 8;4(8):599–604. 12134160 10.1038/ncb826 PMC2387275

[27] Maeda A, Nishida T, Aoyama E, Kubota S, Lyons KM, Kuboki T, et al CCN family 2/connective tissue growth factor modulates BMP signalling as a signal conductor, which action regulates the proliferation and differentiation of chondrocytes. J Biochem 2009 2;145(2):207–16. 10.1093/jb/mvn159 19038999 PMC2760593

[28] Kireeva ML, Latinkic BV, Kolesnikova TV, Chen CC, Yang GP, Abler AS, et al Cyr61 and Fisp12 are both ECM-associated signaling molecules: activities, metabolism, and localization during development. Exp Cell Res 1997 5 25;233(1):63–77. 9184077 10.1006/excr.1997.3548

[29] Nishida T, Kubota S, Aoyama E, Janune D, Maeda A, Takigawa M. Effect of CCN2 on FGF2-induced proliferation and MMP9 and MMP13 productions by chondrocytes. Endocrinology 2011 11;152(11):4232–41. 10.1210/en.2011-0234 21914781

[30] Grotendorst GR, Rahmanie H, Duncan MR. Combinatorial signaling pathways determine fibroblast proliferation and myofibroblast differentiation. FASEB J 2004 3;18(3):469–79. 15003992 10.1096/fj.03-0699com

[31] Grotendorst GR, Duncan MR. Individual domains of connective tissue growth factor regulate fibroblast proliferation and myofibroblast differentiation. FASEB J 2005 5;19(7):729–38. 15857887 10.1096/fj.04-3217com

[32] Kim HS, Nagalla SR, Oh Y, Wilson E, Roberts CT Jr., Rosenfeld RG. Identification of a family of low-affinity insulin-like growth factor binding proteins (IGFBPs): characterization of connective tissue growth factor as a member of the IGFBP superfamily. Proc Natl Acad Sci U S A 1997 11 25;94(24):12981–6. 9371786 10.1073/pnas.94.24.12981 PMC24249

[33] Wang S, Denichilo M, Brubaker C, Hirschberg R. Connective tissue growth factor in tubulointerstitial injury of diabetic nephropathy. Kidney Int 2001 7;60(1):96–105. 11422741 10.1046/j.1523-1755.2001.00776.x

[34] Inoki I, Shiomi T, Hashimoto G, Enomoto H, Nakamura H, Makino K, et al Connective tissue growth factor binds vascular endothelial growth factor (VEGF) and inhibits VEGF-induced angiogenesis. FASEB J 2002 2;16(2):219–21. 11744618 10.1096/fj.01-0332fje

[35] Chen CC, Young JL, Monzon RI, Chen N, Todorovic V, Lau LF. Cytotoxicity of TNFalpha is regulated by integrin-mediated matrix signaling. EMBO J 2007 3 7;26(5):1257–67. 17318182 10.1038/sj.emboj.7601596 PMC1817641

[36] Jun JI, Kim KH, Lau LF. The matricellular protein CCN1 mediates neutrophil efferocytosis in cutaneous wound healing. Nat Commun 2015;6:7386 10.1038/ncomms8386 26077348 PMC4480344

[37] Crean JK, Furlong F, Mitchell D, McArdle E, Godson C, Martin F. Connective tissue growth factor/CCN2 stimulates actin disassembly through Akt/protein kinase B-mediated phosphorylation and cytoplasmic translocation of p27(Kip-1). FASEB J 2006 8;20(10):1712–4. 16790529 10.1096/fj.05-5010fje

[38] Jun JI, Lau LF. Taking aim at the extracellular matrix: CCN proteins as emerging therapeutic targets. Nat Rev Drug Discov 2011 12;10(12):945–63. 10.1038/nrd3599 22129992 PMC3663145

[39] Leask A, Abraham DJ. All in the CCN family: essential matricellular signaling modulators emerge from the bunker. J Cell Sci 2006 12 1;119(Pt 23):4803–10. 17130294 10.1242/jcs.03270

[40] Yoon PO, Lee MA, Cha H, Jeong MH, Kim J, Jang SP, et al The opposing effects of CCN2 and CCN5 on the development of cardiac hypertrophy and fibrosis. J Mol Cell Cardiol 2010 8;49(2):294–303. 10.1016/j.yjmcc.2010.04.010 20430035

[41] Panek AN, Posch MG, Alenina N, Ghadge SK, Erdmann B, Popova E, et al Connective tissue growth factor overexpression in cardiomyocytes promotes cardiac hypertrophy and protection against pressure overload. PLoS One 2009;4(8):e6743 10.1371/journal.pone.0006743 19707545 PMC2727794

[42] Accornero F, van Berlo JH, Correll RN, Elrod JW, Sargent MA, York A, et al Genetic Analysis of Connective Tissue Growth Factor as an Effector of Transforming Growth Factor beta Signaling and Cardiac Remodeling. Mol Cell Biol 2015 6;35(12):2154–64. 10.1128/MCB.00199-15 25870108 PMC4438237

[43] Gravning J, Orn S, Kaasboll OJ, Martinov VN, Manhenke C, Dickstein K, et al Myocardial connective tissue growth factor (CCN2/CTGF) attenuates left ventricular remodeling after myocardial infarction. PLoS One 2012;7(12):e52120 10.1371/journal.pone.0052120 23284892 PMC3527406

[44] Gravning J, Ahmed MS, von Lueder TG, Edvardsen T, Attramadal H. CCN2/CTGF attenuates myocardial hypertrophy and cardiac dysfunction upon chronic pressure-overload. Int J Cardiol 2013 10 3;168(3):2049–56. 10.1016/j.ijcard.2013.01.165 23452880

[45] Szabo Z, Magga J, Alakoski T, Ulvila J, Piuhola J, Vainio L, et al Connective tissue growth factor inhibition attenuates left ventricular remodeling and dysfunction in pressure overload-induced heart failure. Hypertension 2014 6;63(6):1235–40. 10.1161/HYPERTENSIONAHA.114.03279 24688123

[46] Pride YB, Giuseffi JL, Mohanavelu S, Harrigan CJ, Manning WJ, Gibson CM, et al Relation between infarct size in ST-segment elevation myocardial infarction treated successfully by percutaneous coronary intervention and left ventricular ejection fraction three months after the infarct. Am J Cardiol 2010 9 1;106(5):635–40. 10.1016/j.amjcard.2010.04.012 20723637

[47] Pizarro G, Fernandez-Friera L, Fuster V, Fernandez-Jimenez R, Garcia-Ruiz JM, Garcia-Alvarez A, et al Long-term benefit of early pre-reperfusion metoprolol administration in patients with acute myocardial infarction: results from the METOCARD-CNIC trial (Effect of Metoprolol in Cardioprotection During an Acute Myocardial Infarction). J Am Coll Cardiol 2014 6 10;63(22):2356–62. 10.1016/j.jacc.2014.03.014 24694530

[48] Wang QD, Swardh A, Sjoquist PO. Relationship between ischaemic time and ischaemia/reperfusion injury in isolated Langendorff-perfused mouse hearts. Acta Physiol Scand 2001 2;171(2):123–8. 11350272 10.1046/j.1365-201x.2001.00788.x

[49] Reichelt ME, Willems L, Hack BA, Peart JN, Headrick JP. Cardiac and coronary function in the Langendorff-perfused mouse heart model. Exp Physiol 2009 1;94(1):54–70. 10.1113/expphysiol.2008.043554 18723581

[50] Staat P, Rioufol G, Piot C, Cottin Y, Cung TT, L'Huillier I, et al Postconditioning the human heart. Circulation 2005 10 4;112(14):2143–8. 16186417 10.1161/CIRCULATIONAHA.105.558122

